# Supplementary material for: The UbiX flavin prenyltransferase reaction mechanism resembles class I terpene cyclase chemistry
Source: Nat Commun. 2019 May 29;10:2357. doi: 10.1038/s41467-019-10220-1 (PMC6541611; doi:10.1038/s41467-019-10220-1)
Supplement: Supplementary file 1 — Supplementary Information [file 41467_2019_10220_MOESM1_ESM.docx]

**The UbiX flavin prenyltransferase reaction mechanism resembles Class I terpene cyclase chemistry**

Marshall et al.

**Supplementary Information**

|  | *An*UbiX  6QLG | *Pa*UbiX-IMP Reduced  6QLH | W200H *Pa*UbiX-GMP *  6QLV | F181H *Pa*UbiX-DMAP  6QLL | F181H *Pa*UbiX  -DMAP Redox - 6QLK | F181Q *Pa*UbiX-DMAP  6QLJ | F181Q *Pa*UbiX-DMAP Redox  6QLI |
| --- | --- | --- | --- | --- | --- | --- | --- |
| **Data collection** |  |  |  |  |  |  |  |
| Space group | P 4_3_ 2_1_ 2 | F 2 3 | P 2_1_ 2_1_ 2 | F 2 3 | F 2 3 | F 2 3 | F 2 3 |
| Cell dimensions |  |  |  |  |  |  |  |
| *a*, *b*, *c* (Å) | 102.0 102.0 276.7 | a=b=c= 142.1 | 161.5 164.9 102.5 | a=b=c = 142.033 | a=b=c =141.8 | a=b=c 142.5 | a=b=c= 141.9 |
| α, β, γ (°) | α, β, γ = 90 | α, β, γ = 90 | α, β, γ = 90 | α, β, γ = 90 | α, β, γ = 90 | α, β, γ = 90 | α, β, γ = 90 |
| Resolution (Å) | 95.7 - 2.15 (2.227 - 2.15) | 24.02 - 1.57 (1.626 - 1.57) | 115.4 - 2.391 (2.476 - 2.391) | 23.67 - 1.561 (1.616 - 1.561) | 35.44 - 1.78 (1.844 - 1.78) | 42.97 - 1.991 (2.062 - 1.991) | 50.16 - 1.771 (1.834 - 1.771) |
| *R*_merge_ | 0.1247 (0.6224) | 0.1061 (0.7077) | 0.08033 (0.3743) | 0.08613 (0.5909) | 0.08218 (0.696) | 0.09364 (0.6844) | 0.1139 (0.6907) |
| *I* / σ*I* | 15.50 (4.64) | 10.09 (2.08) | 7.02 (2.19) | 8.52 (1.99) | 12.99 (2.24) | 11.64 (2.27) | 9.62 (2.26) |
| Completeness (%) | 99.98 (99.95) | 99.19 (98.01) | 99.98 (99.99) | 98.50 (97.22) | 98.90 (97.91) | 98.97 (97.46) | 98.56 (96.05) |
| Redundancy | 14.7 (14.8) | 6.5 (6.2) | 2.0 (2.0) | 4.3 (4.3) | 6.6 (6.6) | 6.6 (6.6) | 6.6 (6.7) |
|  |  |  |  |  |  |  |  |
| **Refinement** |  |  |  |  |  |  |  |
| Resolution (Å) | 95.7 - 2.15 (2.227 - 2.15) | 24.02 - 1.57 (1.626 - 1.57) | 115.4 - 2.391 (2.476 - 2.391) | 23.67 - 1.561 (1.616 - 1.561) | 35.44 - 1.78 (1.844 - 1.78) | 42.97 - 1.991 (2.062 - 1.991) | 50.16 - 1.771 (1.834 - 1.771) |
| No. reflections | 80280 (7871) | 80280 (7871) | 80280 (7871) | 80280 (7871) | 80280 (7871) | 80280 (7871) | 80280 (7871) |
| *R*_work_ / *R*_free_ | 0.1611/0.1825 | 0.1774/0.1990 | 0.1780/0.2209 | 0.1616/0.1827 | 0.1879/0.2126 | 0.1814/0.2134 | 0.1758/0.2124 |
| No. atoms | 9788 | 9788 | 9788 | 9788 | 9788 | 9788 | 9788 |
| Protein | 8909 | 8909 | 8909 | 8909 | 8909 | 8909 | 8909 |
| Ligand/ion | 289 | 289 | 289 | 289 | 289 | 289 | 289 |
| Water | 590 | 590 | 590 | 590 | 590 | 590 | 590 |
| *B*-factors |  |  |  |  |  |  |  |
| Protein | 33.09 | 33.09 | 33.09 | 33.09 | 33.09 | 33.09 | 33.09 |
| Ligand/ion | 40.43 | 40.43 | 40.43 | 40.43 | 40.43 | 40.43 | 40.43 |
| Water | 39.02 | 39.02 | 39.02 | 39.02 | 39.02 | 39.02 | 39.02 |
| R.m.s. deviations |  |  |  |  |  |  |  |
| Bond lengths (Å) | 0.004 | 0.004 | 0.004 | 0.004 | 0.004 | 0.004 | 0.004 |
|  |  |  |  |  |  |  |  |
| Bond angles (°) | 0.93 | 0.93 | 0.93 | 0.93 | 0.93 | 0.93 | 0.93 |

*Two crystals were used for W200H *Pa*UbiX-GMP data collection

**Supplementary Table 1: X-ray crystallography data and refinement statistics**


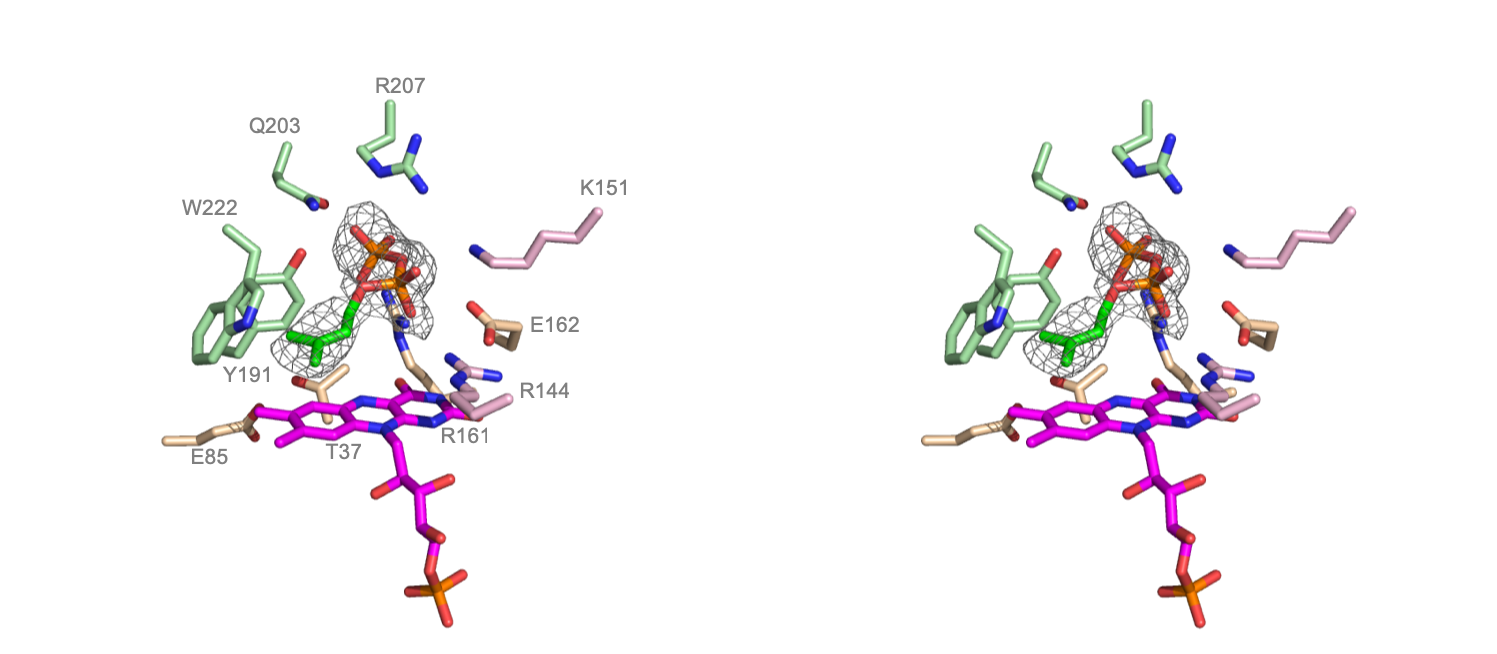


**Supplementary Figure 1: Stereo image of Fig 2b.** Crystal structure of *An*UbiX in complex with FMN and DMAPP. The *An*UbiX active site is shown with the omit polder map corresponding to bound DMAPP contoured at 3 sigma. Residues are coloured according to different *An*UbiX monomers.


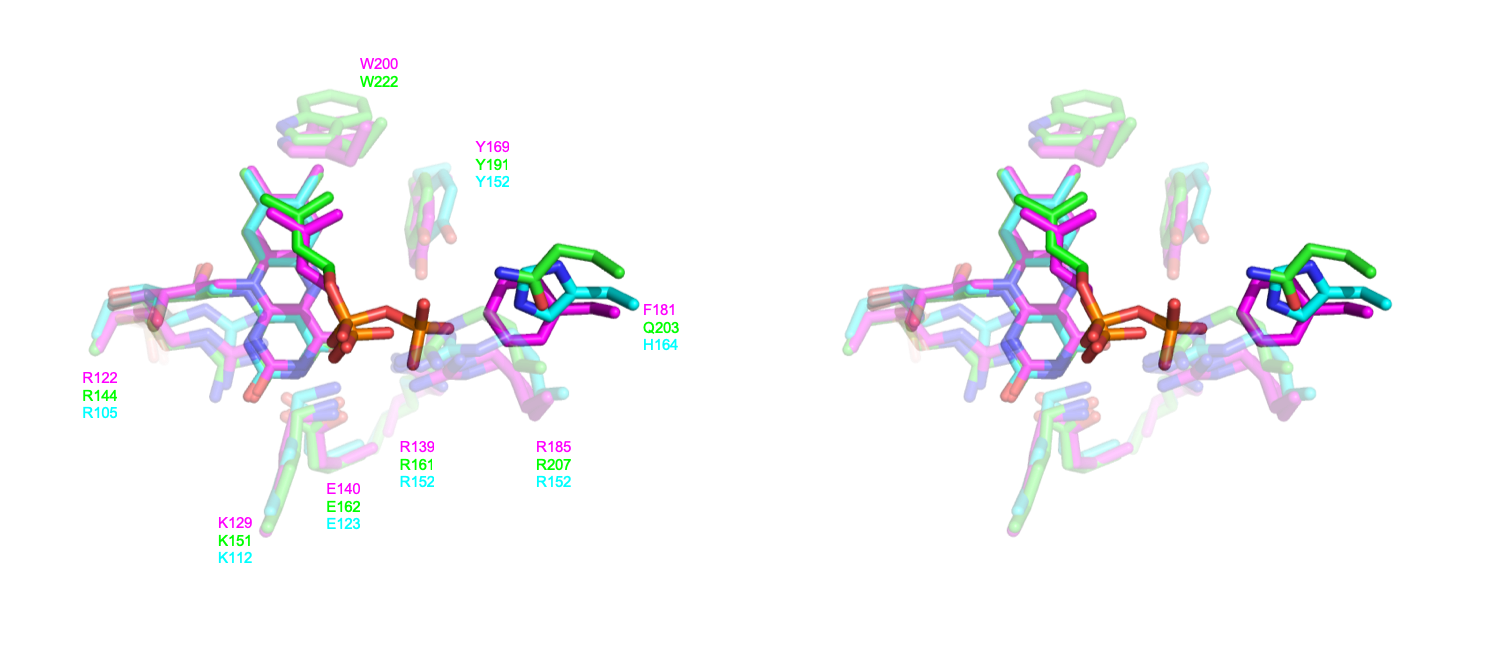


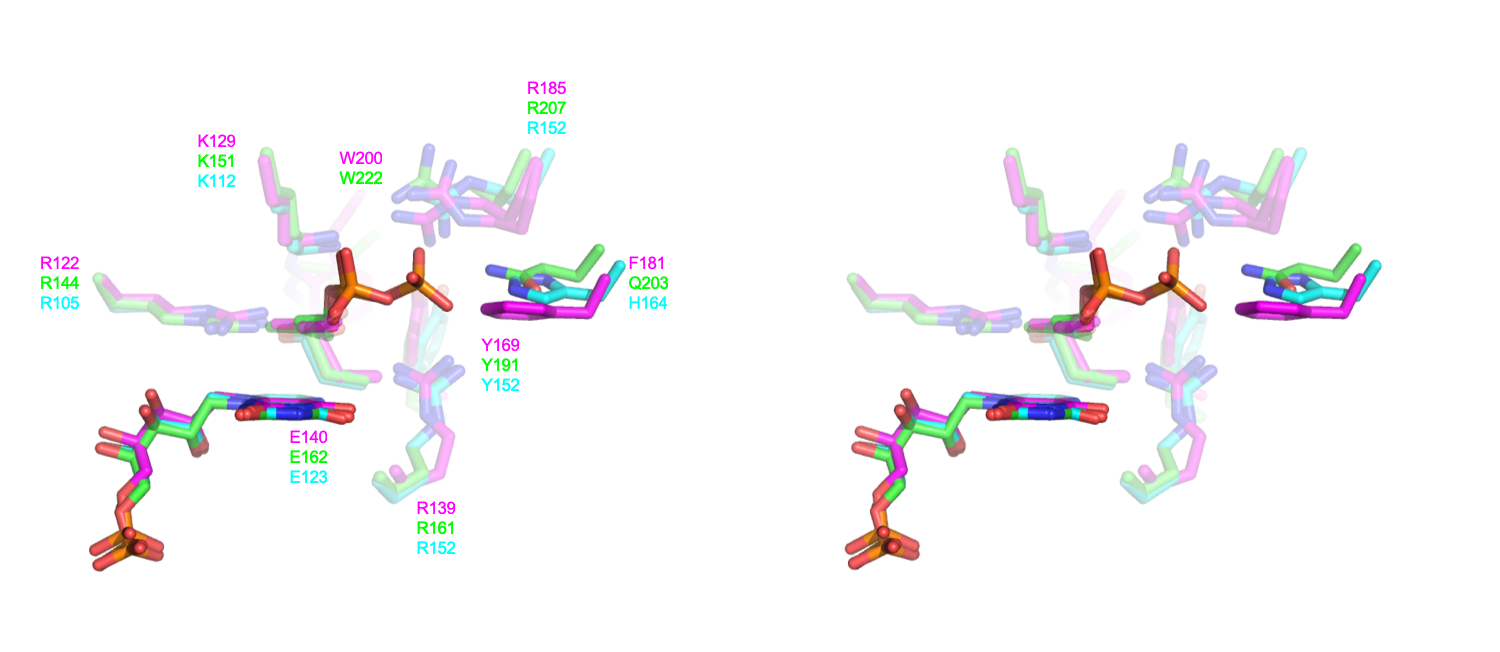


**Supplementary Figure 2: Stereo image of Fig 2c.** A structural overlay of *Pa*UbiX (magenta), *An*UbiX (green) and *O157*UbiX (cyan) active sites. This reveals a highly conserved network of substrate binding residues. Bottom image is a 90° rotation about the X-axis.

**Supplementary Figure 3: Structure of phosphorylated compounds used for binding studies.** DMAP, geranyl monophosphate, pentenyl phosphate and 3-methylbutyl phosphate were synthesised as described. DMAPP, IMP and 2-ethylhexyl phosphate were purchased from Sigma.


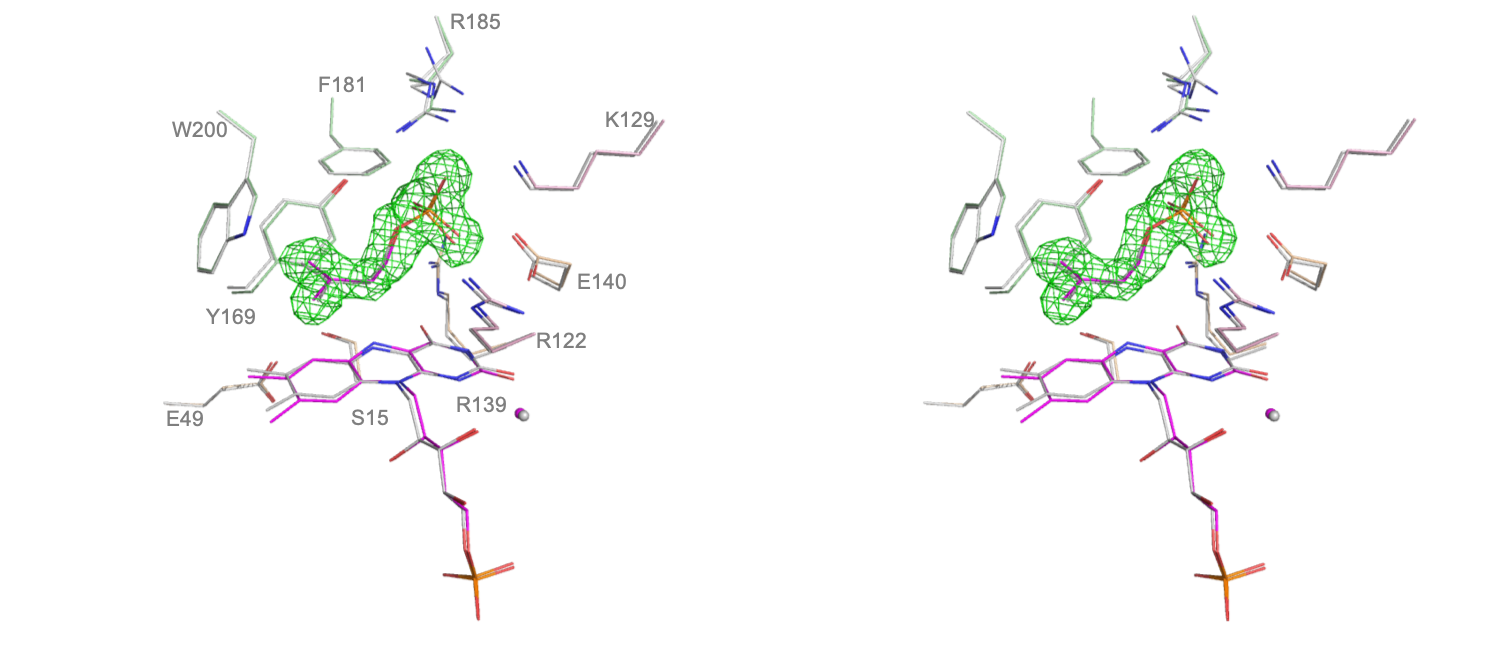


**Supplementary Figure 4: Stereo image of Fig 3b.** Crystal structure of the *Pa*UbiX : FMNH_2_ : IMP complex superposed with previously determined *Pa*UbiX : FMN : DMAP (4ZAF) in grey. The omit polder map corresponding to bound IMP is shown in green mesh contoured at 5 sigma. Residues are coloured according to *Pa*UbiX monomer.


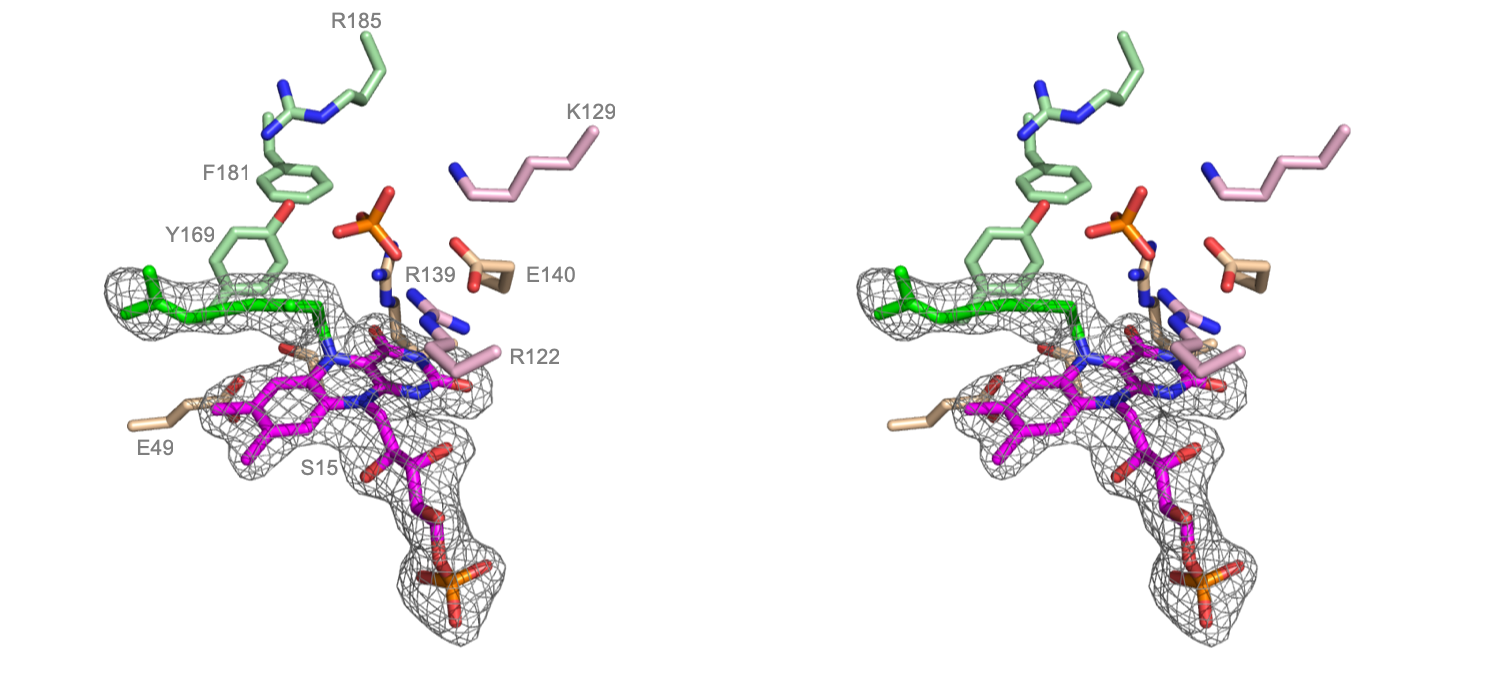


**Supplementary Figure 5: Stereo image of Fig 3d.** Crystal structure of *Pa*UbiX W200H with an N5 geranyl adduct (green) following incubation of reduced crystals with geranyl monophosphate. Omit polder map corresponding to the bound geranyl N5 FMN shown in grey mesh contoured at 4 sigma. The W200H mutation is not visible due to disorder of the C-terminus.


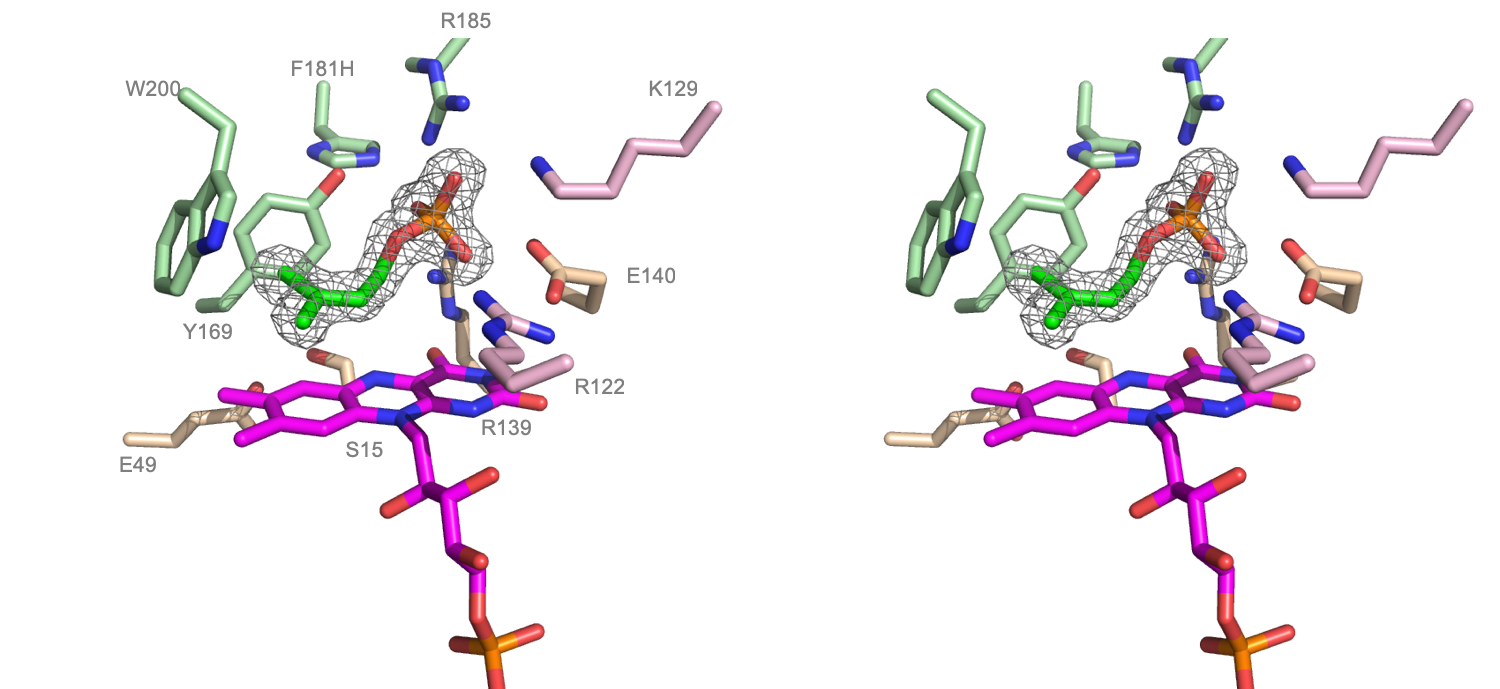


**Supplementary Figure 6: Stereo image of Fig 4a.** Crystal structure of the F181H *Pa*UbiX variant in complex with DMAP and FMN. Omit polder map corresponds to bound DMAP contoured at 3 sigma.


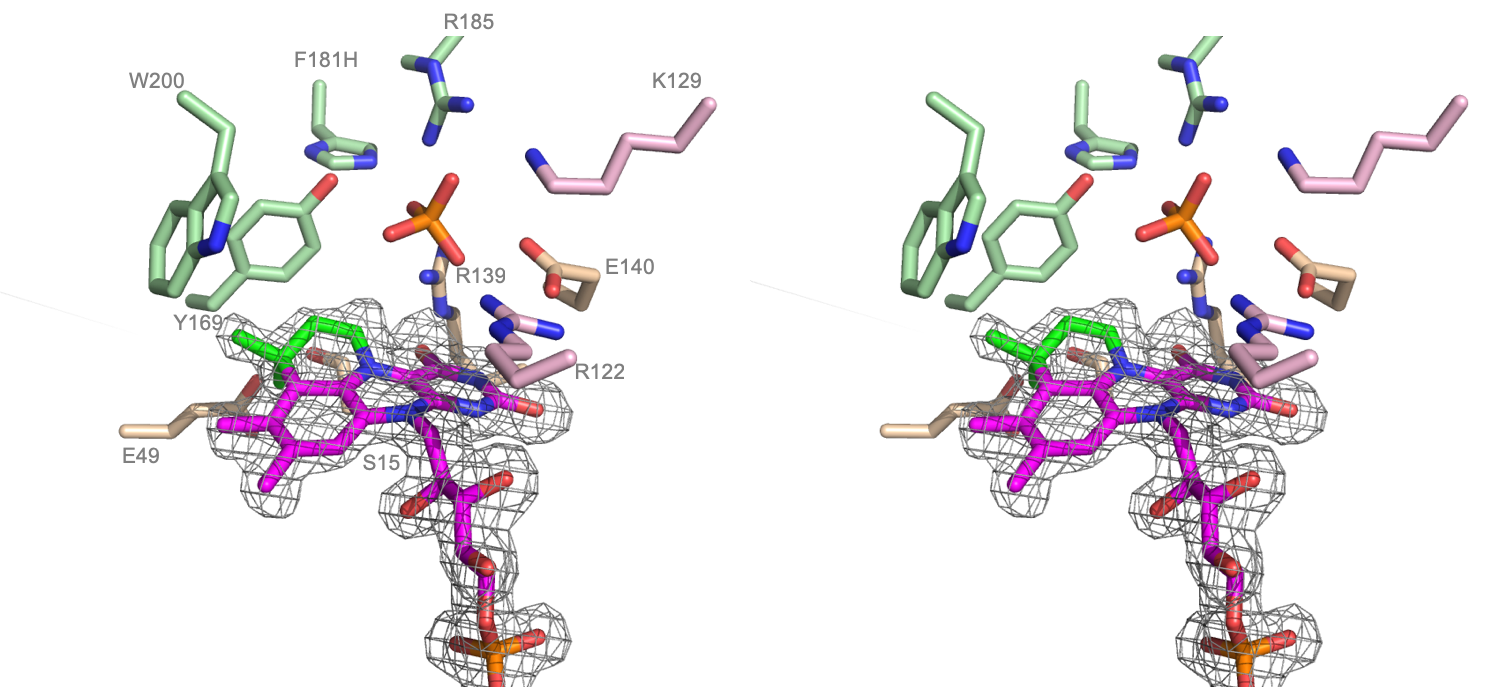


**Supplementary Figure 7: Stereo image of Fig 4b.** Crystal structure of F181H *Pa*UbiX variant crystals following reduction and subsequent reoxidation revealing formation of prFMN. Omit polder map corresponds to bound prFMN contoured at 3 sigma.


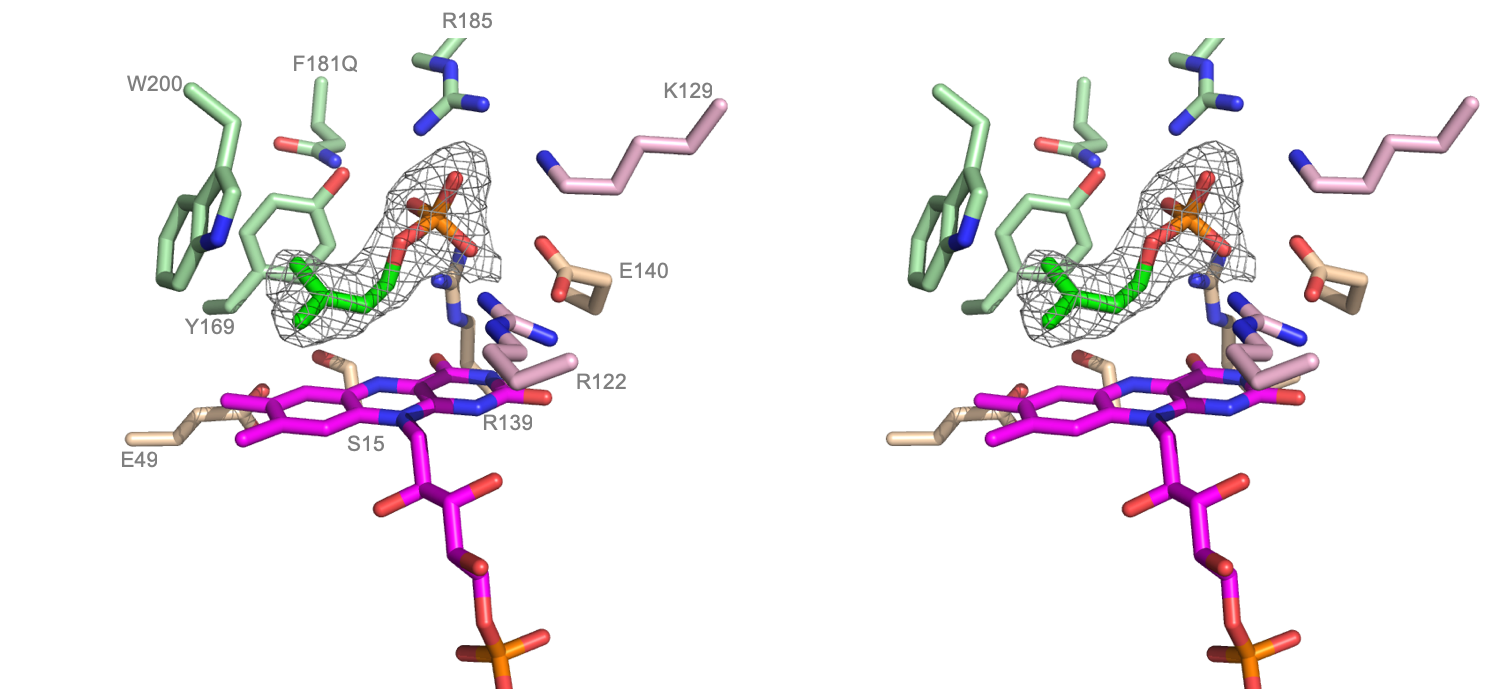


**Supplementary Figure 8:** **Stereo image of Fig 4c.** Crystal structure the F181Q *Pa*UbiX variant in complex with DMAP and FMN. Omit polder map corresponds to bound DMAP contoured at 3 sigma.


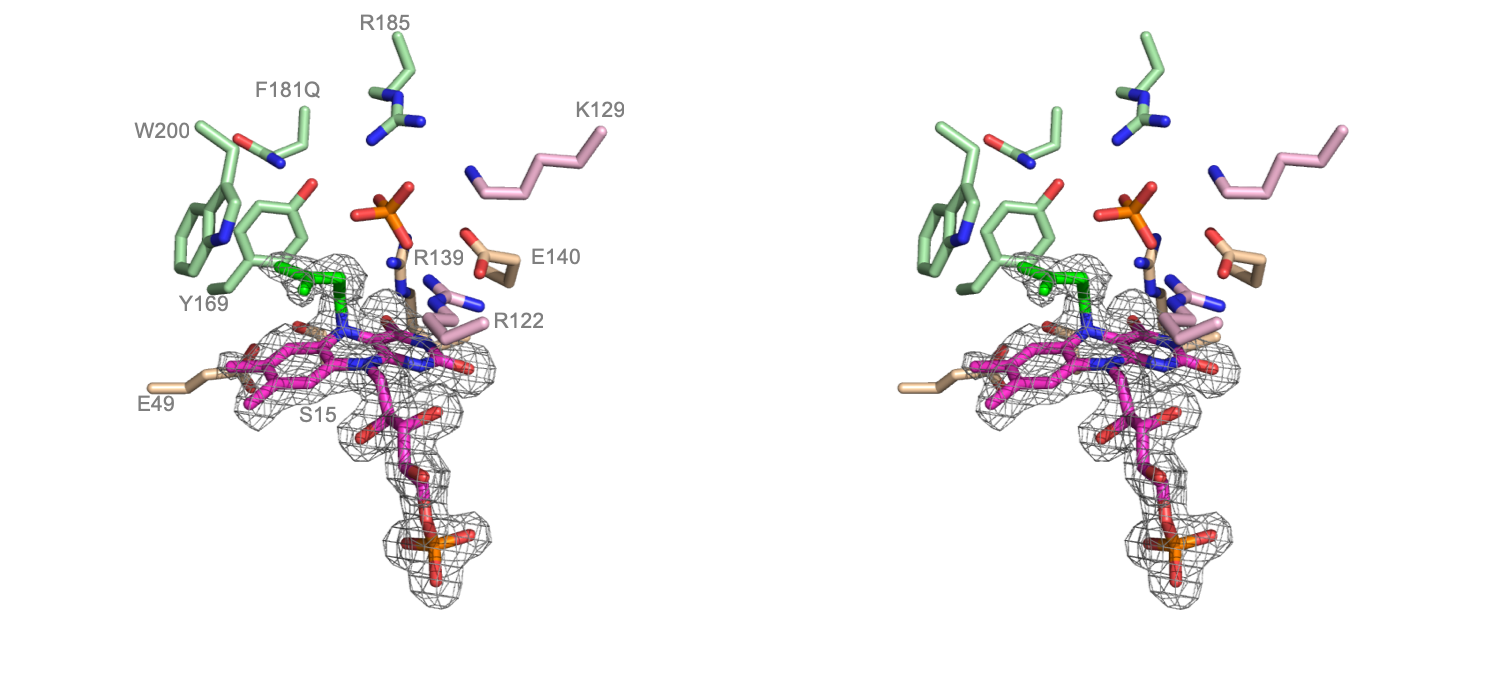


**Supplementary Figure 9: Stereo image of Fig 4d.** Crystal structure of F181Q *Pa*UbiX variant crystals following reduction and subsequent reoxidation revealing the reaction does not proceed beyond N5-prenylation in this variant. Omit polder map corresponds to bound FMN adduct contoured at 3 sigma.


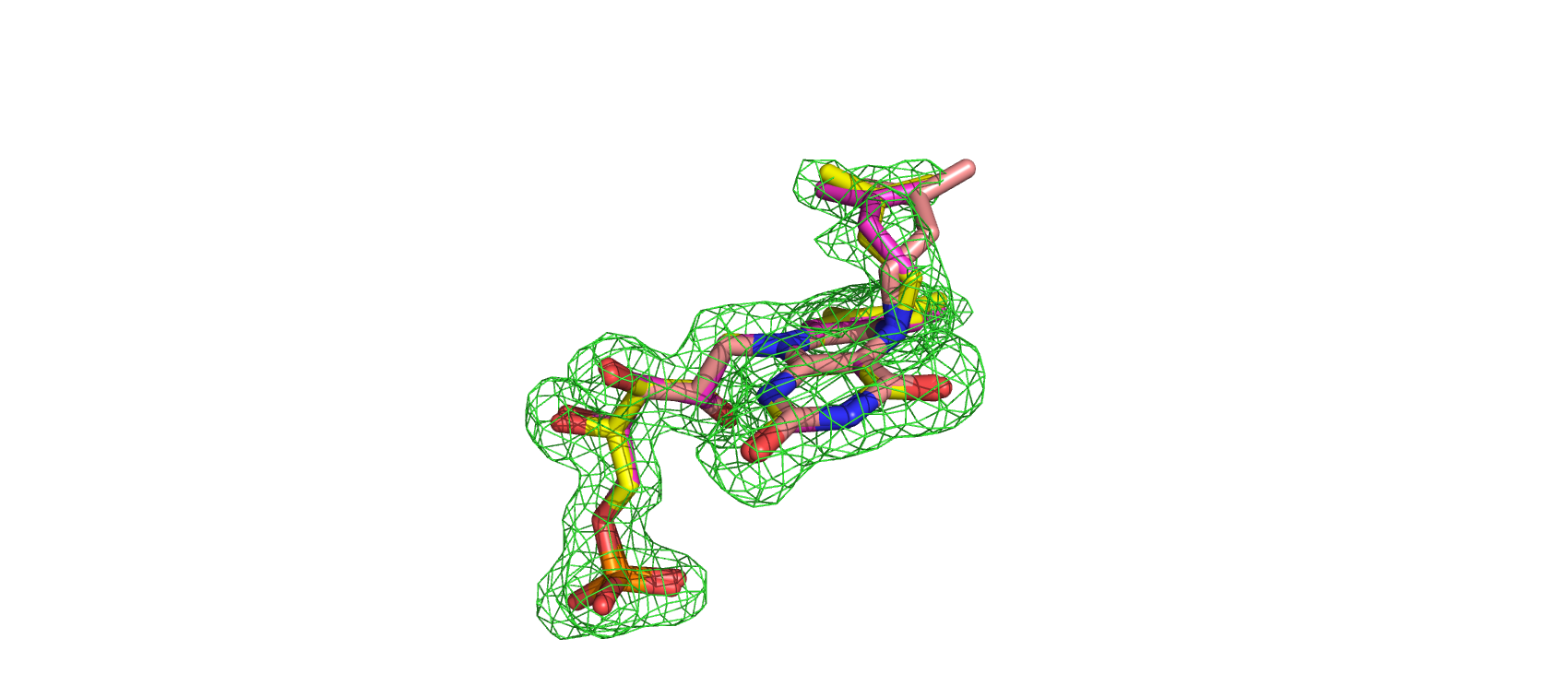


**Supplementary Figure 10: Overlay of the N5-dimethylallyl adduct structure in distinct *Pa*UbiX variants.** The omit polder map corresponding to the F181Q variant is shown in a green mesh contoured at 3 sigma (magenta - F181Q; yellow - Y169F, salmon - E49Q). The F181Q dimethylallyl conformation is similar to that previously observed for Y169F, and distinct from the previously observed E49Q conformation.


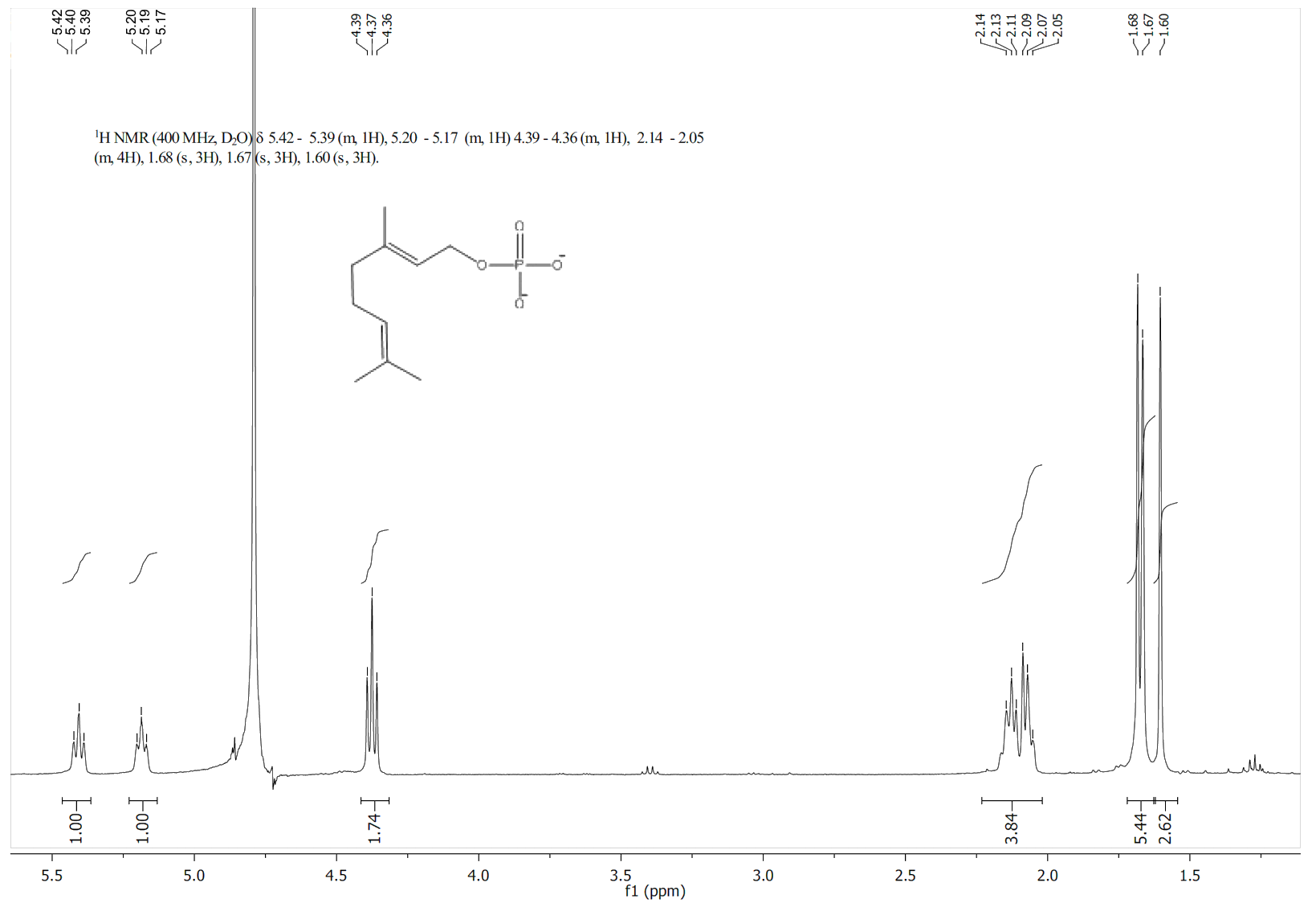


**
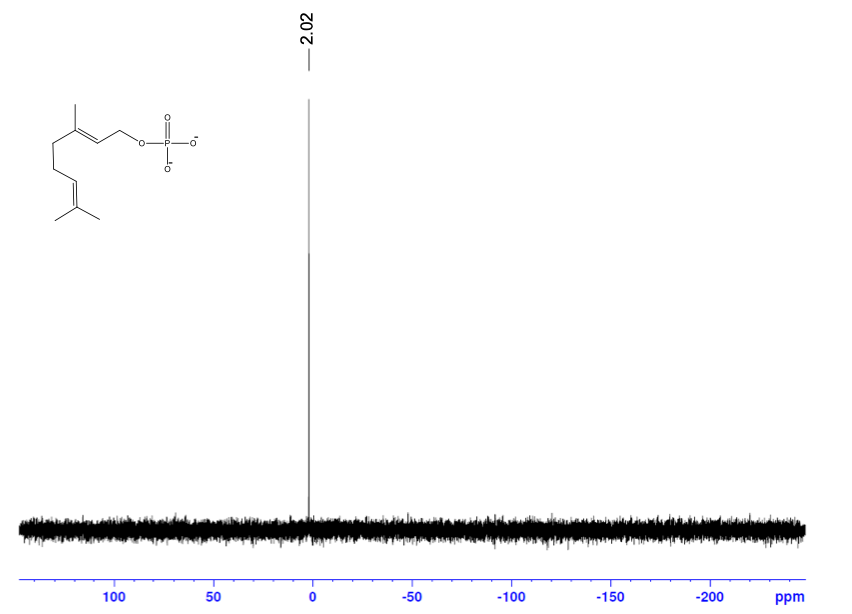
**

**Supplementary Figure 11:** ^1^H NMR and ^31^P NMR of geranyl monophosphate (GMP)

^1^H NMR (400 MHz, D_2_O) δ 5.42 - 5.39 (m, 1H), 5.20 - 5.17 (m, 1H), 4.39 - 4.36 (m, 1H), 2.14 - 2.05 (m, 4H), 1.68 (s, 3H), 1.67 (s, 3H), 1.60 (s, 3H)

^31^P NMR (162 MHz, D_2_O) δ 2.02


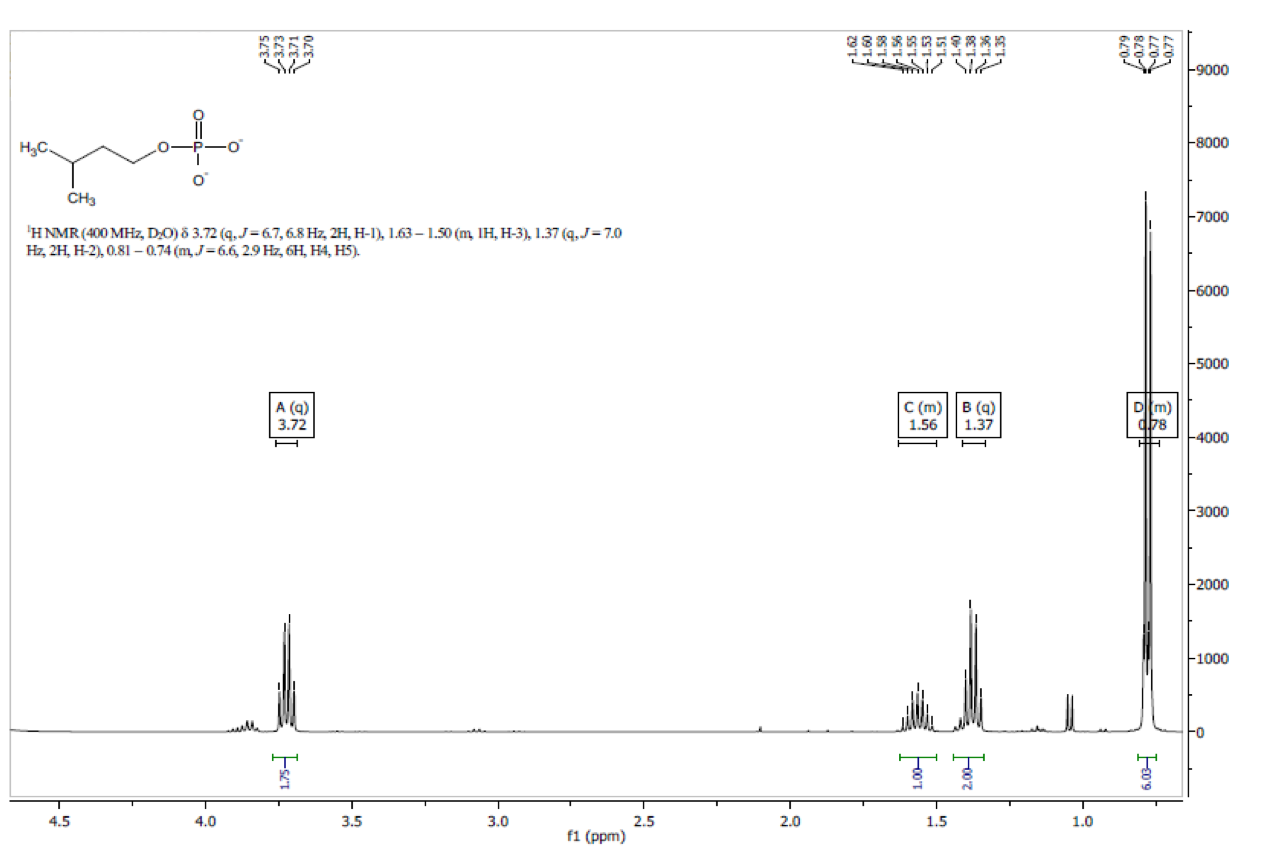


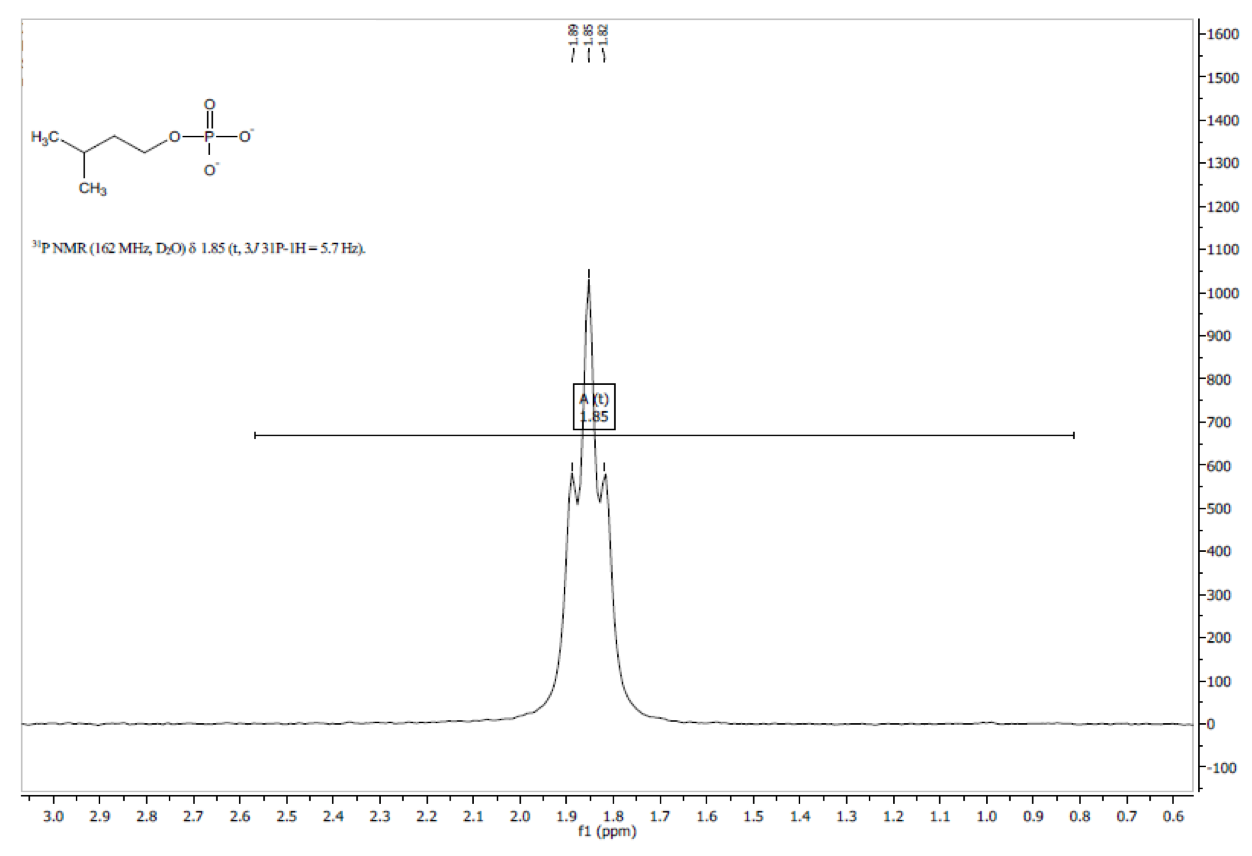


**Supplementary Figure 12:** ^1^H NMR and ^31^P NMR of 3 – methylbutanyl monophosphate

^1^H NMR (400 MHz, D_2_O) δ 3.72 (q, *J* = 6.7, 6.8 Hz, 2H), 1.63 – 1.50 (m, 1H), 1.37 (q, *J* = 7.0 Hz, 2H), 0.81 – 0.74 (m, *J* = 6.6, 2.9 Hz, 6H)

^31^P NMR (162 MHz, D_2_O) δ 1.85 (t, ^3^*J*^31^P-^1^H = 5.7 Hz)


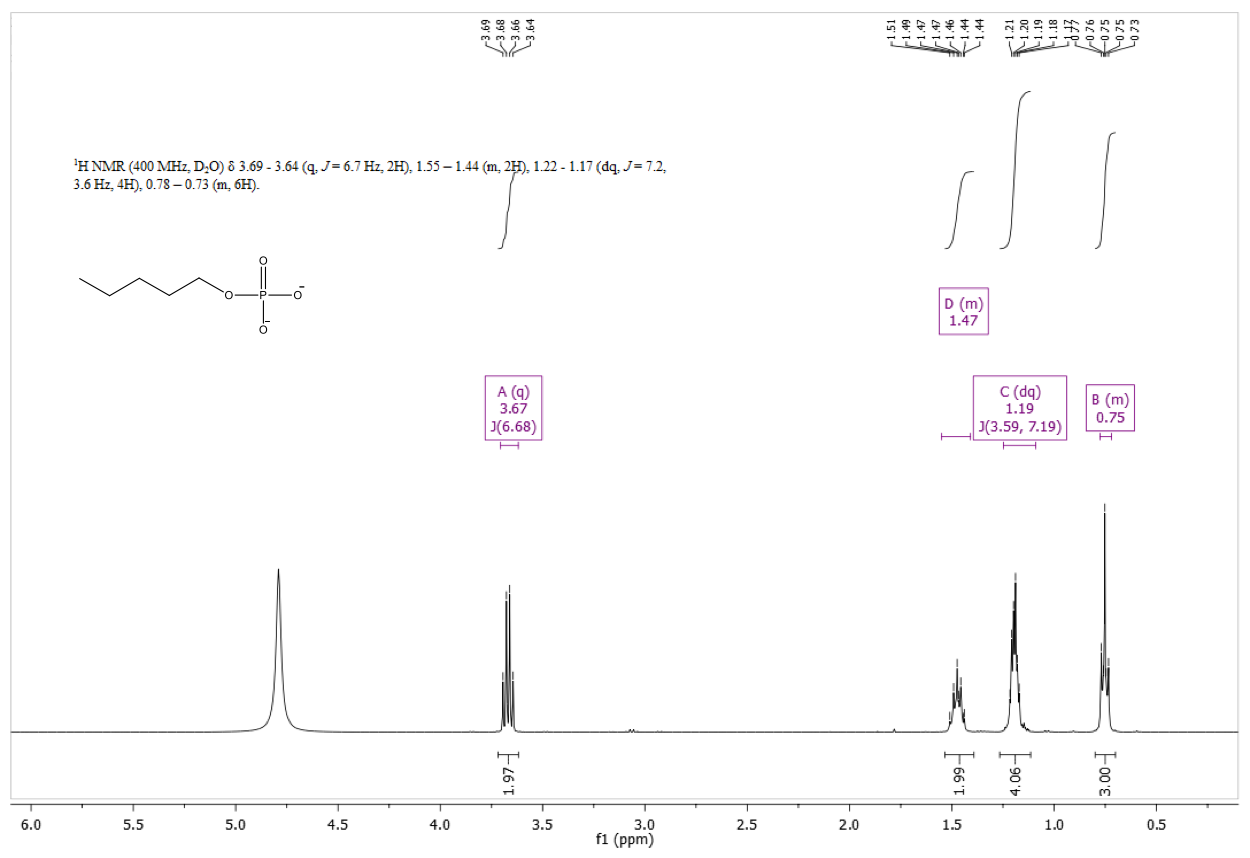


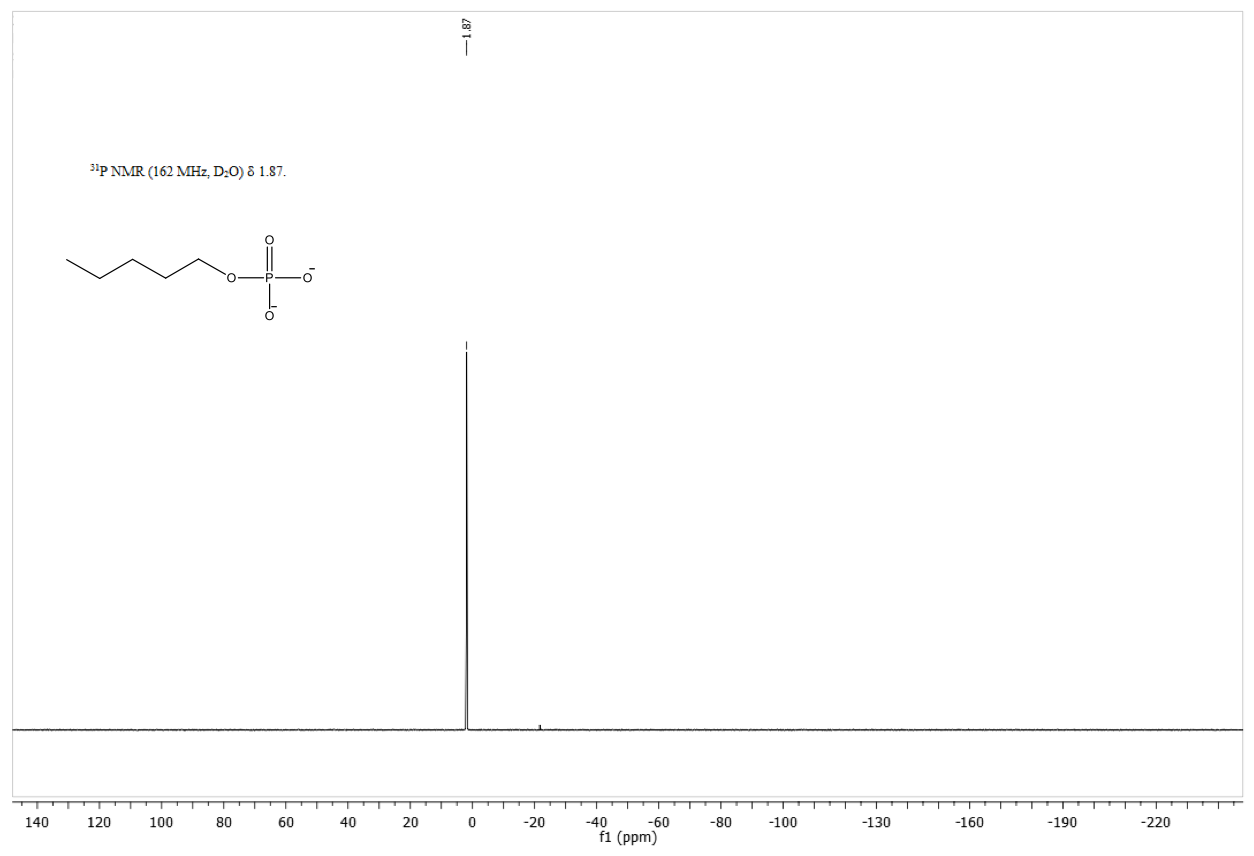


**Supplementary Figure 13:** ^1^H NMR and ^31^P NMR of pentanyl monophosphate

^1^H NMR (400 MHz, D_2_O) δ 3.69 - 3.64 (q, *J* = 6.7 Hz, 2H), 1.55 – 1.44 (m, 2H), 1.22 - 1.17 (dq, *J* = 7.2, 3.6 Hz, 4H), 0.78 – 0.73 (m, 6H)

^31^P NMR (162 MHz, D_2_O) δ 1.87
